# An Artificial Intelligence of Things-Based Picking Algorithm for Online Shop in the Society 5.0’s Context

**DOI:** 10.3390/s21082813

**Published:** 2021-04-16

**Authors:** Muslikhin Muslikhin, Jenq-Ruey Horng, Szu-Yueh Yang, Ming-Shyan Wang, Baiti-Ahmad Awaluddin

**Affiliations:** 1 Department of Electrical Engineering, Southern Taiwan University of Science and Technology, Tainan 710, Taiwan; da72b206@stust.edu.tw (M.M.); jrhorng@stust.edu.tw (J.-R.H.); Da720201@stust.edu.tw (S.-Y.Y.); da82b207@stust.edu.tw (B.-A.A.); 2 Department of Electronics Engineering Education, Universitas Negeri Yogyakarta, Yogyakarta 55281, Indonesia

**Keywords:** artificial intelligence of things, data-driven mode, deep learning, industry 4.0, modified YOLOv2, society 5.0

## Abstract

In this study, an Artificial Intelligence of Things (AIoT)-based automated picking system was proposed for the development of an online shop and the services for automated shipping systems. Speed and convenience are two key points in Industry 4.0 and Society 5.0. In the context of online shopping, speed and convenience can be provided by integrating e-commerce platforms with AIoT systems and robots that are following consumers’ needs. Therefore, this proposed system diverts consumers who are moved by AIoT, while robotic manipulators replace human tasks to pick. To prove this idea, we implemented a modified YOLO (You Only Look Once) algorithm as a detection and localization tool for items purchased by consumers. At the same time, the modified YOLOv2 with data-driven mode was used for the process of taking goods from unstructured shop shelves. Our system performance is proven by experiments to meet the expectations in evaluating efficiency, speed, and convenience of the system in Society 5.0’s context.

## 1. Introduction

It is quite challenging to ensure that the massive implementation of artificial intelligence (AI) in online shopping takes effect. Currently, most of AI dominated systems are implemented in large-scale industries, such as Amazon, Walmart, BMW, and Mercedes Benz [1,2,3]. Separately, Industry 4.0 including techniques of AI, Internet of Things (IoT), robots, and online shopping has now shown tremendous benefits. Especially during the COVID-19 pandemic like today, physical contact is much avoided. In other words, the role of Industry 4.0 content should be accelerated to meet the needs of people who tend to prioritize comfort and safety.

Since Society 5.0 was introduced in Japan, the role of IoT has become the backbone of the related system [4]. In addition to the emergence of AI in the last decade, the appearance of IoT and AI gives rise to the terminology, Artificial Intelligence of Things (AIoT), which demonstrates the basic principle of how to think of a device like a human with the support of an internet network [5,6,7]. Meanwhile, Society 5.0 takes the maximum benefit from AIoT in daily life [8,9]. Many realities are in line with the concept of Society 5.0, such as shopping. However, shopping has experienced several downsizings, for instance, in online shopping, order delivery using applications, shopping with electronic payments, limited shopping served by machines (vending machines), and online shopping facilitated by AIoT. Unfortunately, the integration of AIoT in online shopping has not been widely reported.

Some of the AIoT approaches work for other applications [10]. Two studies [11,12] have introduced AIoT for drug screening applications and hair health diagnostics. Including further research, MedGlasses helps visually impaired patients to take medication easily [13]. Still, [14] used deep learning and IoT to detect traffic accidents within 7 s detection response. Another implementation was offered by [15] to recognize the emotional expression of students on campus to be emotionally aware, humanist, and as personal assistants. The system was developed using a Deep Neural Network (DNN) and the AlexNet was introduced [16]. Furthermore, [17] proposed the faster region-based convolutional neural network (R-CNN), which has better speed also accuracy than AlexNet, CNN, R-CNN, and Fast R-CNN. Another quite capable method is offered by [18] with the YOLO (You Only Look Once) method, the last being YOLOv3. As for YOLOv2, the detection speed is still superior to the Faster R-CNN.

YOLO continues to be refined and applied in other fields: robotics, automotive, medicine, and military. The YOLOv3 technique with a modification to curtail floating point operation (FLOP) was modified by [19], which conducted a performance increase 250% faster. YOLOv3 also uses K-means in each target to distinguish the clusters, and provides the accuracy more than 90% [20]. However, YOLOv3 requires a fast GPU and computer, implying that not only high specification hardware is needed, but also some detection approaches solely focus on the confidence value. In robotic applications, the grasping process is essential after the confidence level is known [21,22].

To achieve the reliability and validness, the system needs to localize a target after recognition and detection. Several techniques to achieve this goal use a mono camera, stereo camera, or depth camera. An RGB-D camera is prevalent among them, but its construction is still a little bigger than a mono camera [23]. Especially, it will be an obstacle when entering narrow gaps if the eye-in-hand mode is used. The standard Hough Transform image processing technique was employed by [24] to identify the environmental classification of strawberries. References [24,25,26] also use RGB-D images to estimate targets and perceive obstacles in each environment.

In order to ensure the safe picking of products in a shop shelf setting, deep estimation is crucial for industrial robots. The use of FRAMOS, MultiSense, ZED, RealSense, or Kinect, an RGB-D camera is not recommended because it has a relatively larger size than that of a mono camera. On the other hand, narrow shelf positions, the random product ordered, orientation, varying dimensions, and different types of packing are important objectives other than depth estimation [8,9,27].

References [28,29,30] focused on AI, robots, and data mining for online shops. Next, [28] analyzed consumer behavior of various attributes, gazing, listening, and smelling when shopping on online sites. While [29] used the particle swarm optimization (PSO) technique to package online shopping products; unfortunately, the study did not discuss the details of its online shop. Meanwhile, [30] tends to data mining, which is presented in a preference matrix so that within a specific time it can be seen which products are selling well. Both of [28] and [30] used the online shop platform with IoT and data mining implementation. Still, it did not explain how the service process on the offline shop side works, and we suspected that the goods collection system has not been integrated with robots. Furthermore, [31] had involved online shop platforms with robots, however, the process of taking goods from the shop shelves still involved humans; hence, there may be a chance of an error occurring before passing on to the packaging section.

In our prior work [23], object localization and depth estimation had implemented the R-CNN on eye-in-hand construction using a mono camera, which recognized and sorted the target and then estimated the target’s depth (*Z*) and centroid. At the same time, the other pair coordinates (*XY*) are obtained from the left camera and right camera disparity. In the additional work [32], a stereo camera was also employed for picking using CNN with eye-to-hand structure. Nevertheless, the applications on the target are relatively easy to recognize almost uniform shapes and colors even in piled up and cluttered environments. Our challenge lies in a vertical environment because of the use of a single camera acting as a stereo camera. So, the points of disparity will be grouped by several approaches [33]. From the online shop side, the representational state transfer application programming interface (REST API) reply time and internet data traffic density are obstacles for the system [34]. These works focus on image processing, AIoT combined with industrial robots picking exactly according to products purchased by consumers. In this paper, the pick-up accuracy and the speed of the retrieval service from a stacked shop shelf environment will be focal points.

Online shopping services for consumers and from the offline shop side are proposed and completed. In this paper, the following details are given:

- The REST API is operated to get a reply from the online shop, which depends on the last transaction, and a selective data-driven mode completed by “data/last_transaction” data for YOLOv2 is proposed.
- The shelf collision obstacle for manipulators in shop shelves is weighed. This problem is solved by proposing a modified selective YOLOv2 technique to classify the edge of shelf as a forbidden points cloud to avoid each shelf edge.
- We are specific to robotic manipulator conditions, and the AIoT-based picking algorithm is implemented and evaluated; it provides a reference for eye-in-hand manipulator systems concerning Society 5.0 in terms of comfort and safety.

In this paper, Section 2 addresses the system design. Section 3 acquaints an online shopping platform until the system sending the last transaction to the offline shop, and the robot with AIoT based picking algorithm would execute it in Section 4. Experimental results are shown in the next Section 5. Finally, the work and give suggestions for potential future work are concluded in Section 6.

## 2. System Design

Our system consists of two parts; online shop platforms and offline shops equipped with manipulator robots. The online shop provides functions that, as usual, consumers could shop based on the App or website. Before being able to shop, potential customers must register first, including name, mobile number, address, city, and zip code. After buying and completing payments, consumers are asked to upload proof of payment. On the online shop, an admin would not verify because of our system set as a data-driven mode. In the offline shop side, the manipulator robot with an eye-in-hand structure will receive a REST API request-reply shortly after the administrator validates the payment. One data packet is sent to the client’s PC and the robot grasps the purchased products.

**Figure 1:**
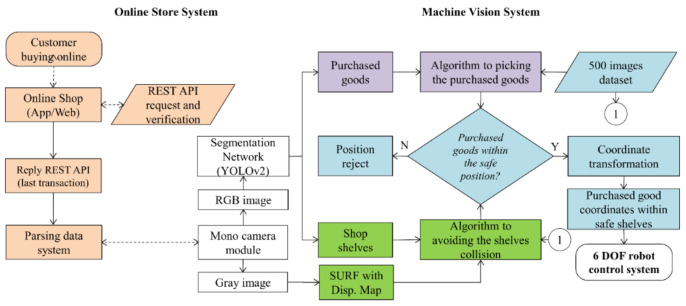
Overall architecture diagram of the AIoT based picking algorithm.

The architecture of the proposed AIoT is shown in Figure 1. Instance segmentation in the YOLOv2 network is used to identify products that are stacked on a shelf after the recognized product undergoes a safe operation check-in XYZ coordinates and a collision side detection algorithm with deep learning to obtain an accurate and secure final position.

The proposed algorithm involves identifying the product as well as picking the obstacle edges of the shop shelf environment in the 2D drawing. In Figure 1, the green blocks refer to the selective localization of products, while those shaded in orange correlate with the online shop platform and the white shades represent the deep learning process. These two goals coordinate with each other to complete the product picking mission so that the gripper avoids collision; hence, the procedures associated with both objectives are shaded in cyan. The picking algorithm would be explained and determined in Section 4.

## 3. Online Shop in Society 5.0

### 3.1. Online Shop

A website-based online shop has developed, as at https://indoaltantis.com (accessed on 15 October 2020). This online shop includes three kinds of users: customers, staff, and administrator. To fulfill the App version, we convert it to an app form to access the Android or iOS platforms.

The minimal features in the online shop are applied because AIoT for picking algorithms are focused and developed. These features include displaying offers, payments, verification, and stock updates. As previously mentioned, potential customers must register in shop and be served by the system. This online shop uses the Laravel PHP framework with a MySQLi database and has adopted the transport layer security (TLS) protocol for its standard security.

**Figure 2:**
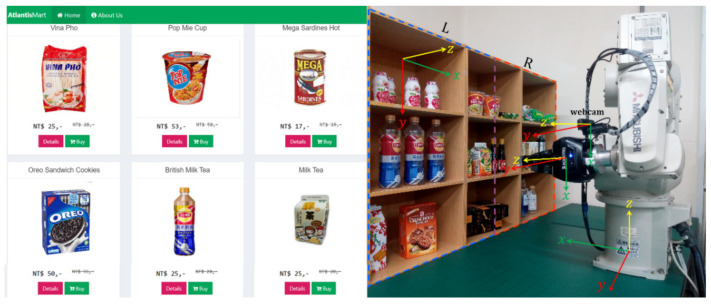
The online (**left**) and offline shop with a 6 degree-of-freedom (6 DOF) robotic manipulator (**right**).

The characteristics of the products sold at our shop vary widely, ranging from different size, weight, packaging type, and packaging form. This needs to consider that every object has a different treatment. For example, taking instant noodles is different from taking sardines cans. Details of the products are presented in Table 1. In addition to viewing the purchased products on the proposed Android-based mobile device app, a customer’s records stored on the cloud-based management platform can also be visited through a website, as shown in Figure 2.

### 3.2. Society 5.0′s Context

The context of Society 5.0 [34] is specifically a way to achieve Sustainable Developing Goals (SGDs) in which it is composed of several cutting-edge technologies. The technologies include 17 basic techniques such as; IoT, big data, AI, drone, robot, 3D print, sensor, sharing on demand, mobile, edge, cloud, 5G, public key infrastructure (PKI), virtual reality (VR), augmented reality (AR), and mixed reality (MR). They can be grouped into two parts; information and cyber-physical system (execution), like a coin with two sides; accuracy and speed.

Accuracy is closely related to precision. There are four probabilities: accurate-precise, accurate-unprecise, inaccurate-precise, and inaccurate-unprecise in testing. In the system, accuracy leads to recognition, localization, and retrieval by robots. Whereas speed refers to the duration of the REST API reply, the speed at which the purchased product is recognized, and the overall retrieval by the system [35,36].

The key to implementing Society 5.0 lies in Science, Technology, and Innovation (STI), creating new value through Cyber-Physical System (CPS), and multidisciplinary collaboration as we apply in this study [35]. The results of the accuracy and speed evaluation are specifically discussed in Section 5.

### 3.3. AIoT with Data-Driven Mode

The website and app versions of the online shop have been developed, although data communication with the offline shop is necessary. In this process, we take advantage of the REST API that is a communication architecture using the HTTP protocol for data exchange [31]. Meanwhile, the web service API works at the operating system level to help applications communicate with the base layer and with each other according to protocols.

**Figure 3:**
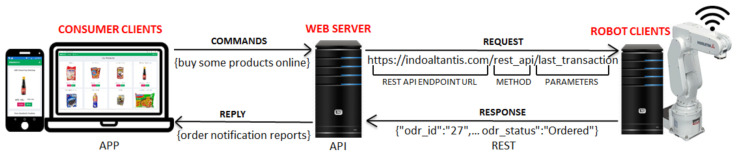
The AIoT with REST API mechanism, each request would be responded to a set of the last transaction data.

The reply to the REST API request on our web server contains explicit details of the purchase of goods. The raw data for item purchases include 27 parameters to be parsed according to the minimum requirements for robot tasks in offline shops. The primary data contain the buyer name, the product name, the purchased quantity, the full address, the zip code, and the telephone number. The REST API mechanism is seen in Figure 3.

**Figure 4:**
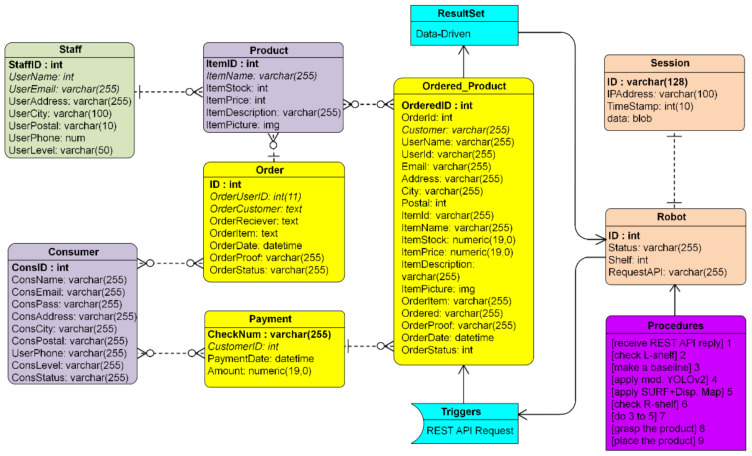
The ERD of the whole database in an online shop with REST API request and data-driven mode.

Broadly, Figure 3 reveals that the data flow from the customer to the server was forwarded to the client robot. In fact, the client robot is not directly connected to the internet by a wire or wireless connection, but there is a middleware in the form of a controller that connects an internet computer. On the server-side, it is as much as possible designed lightly with page size < 5 MB and reply speed < 5.3 s. These require an entity-relationship diagram (ERD), as shown in Figure 4. Eight items in databases, staff, consumer, product, order, payment, ordered product, robot, and session, are connected. With the session containing the IP address system, it is designed to support observing the location of robots/offline shops in a map distribution.

### 3.4. Purchased Products Recognition

**Figure 5:**
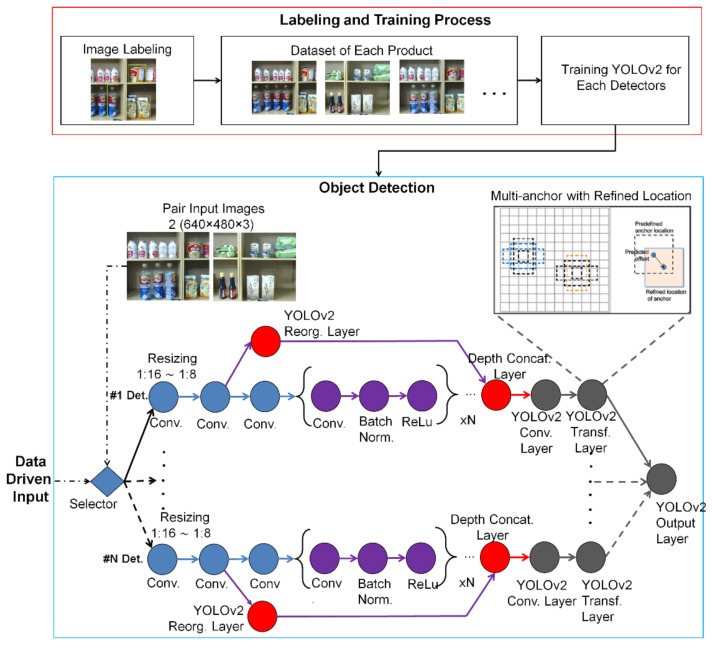
Data-driven mode of YOLOv2 with selective detector. The #1 Det is order of detectors which depend on kind of the products in the shop. Upper box (red) is labeling process for each detector in training stage. Lower box (blue) is parallel detector by data-driven mode in detection stage of the modified YOLOv2.

The CNN, RCNN, Fast RCNN, and Faster RCNN are trained to perform bounding box classification and regression simultaneously [23]. In practice, YOLO runs much faster than Faster RCNN due to its simpler architecture. YOLO architecture is inspired by GoogleNet, see Figure 5. As explained in the introduction, various detection techniques are valid, reliable, and fast. Recognition results are generally followed up with grasping or other commands [16,17,18,32,37]. Moreover, the estimation of the purchased product position in 3D space is expected to be as validly as possible.

Although being one of the most popular recognition methods, YOLOv3 [20,38] not only has heavy computation but also needs the GPU with the highest computing ability for Compute Unified Device Architecture (CUDA). Because of this development to be implemented in small and medium enterprise (SME) shops, we decided to utilize the onboard GPU, and YOLOv2, not YOLOv3. Even though using YOLOv2, we modify YOLOv2 to have the capability of being fast and accurate from the original version. We apply a data-driven mode for the selected detector. In short, with only specific anchors used, there is an opportunity to carry out the recognition process twice, so that the product depth validation on shop shelves can be reached by the robot.

To realize the purpose of recognizing purchased products, the YOLOv2 needs a training stage like in common deep learning. In the training process, image datasets are labeled by the MATLAB Image Labeler App. All have nine classes of RoI (Regions of Interests) namely ABC Ketchup, British Milk Tea, Gao Vermicelli, Instant Cup Noodle, Apple Yogurt, Soto Noodle, Lageo Wafers, Master Sardines, and Tai Lemon Tea generated from around 1500 images.

The YOLOv2 feature extraction layer is the most effective when the output feature width and height are between 8 and 16 times smaller than those of the input image. This amount of downsampling is a trade-off between spatial resolution and performance quality characteristics. Note that selecting the optimal feature extraction layer requires empirical evaluation. This means that labeling requires accuracy. To identify object classes in an image, YOLOv2 uses anchor boxes and predicts these three attributes for each anchor box; (1) intersection over union (IoU) predicts each anchor box’s objectivity score, (2) the anchor box offsets an improvement to the location of the anchor box, (3) probability of class predicts the class label assigned to each anchor box.

The YOLOv2ReorgLayer function in Figure 5 generates a YOLOv2ReorgLayer object that shows the reorganization layer for the YOLOv2 object detector network. By piling adjacent characteristics into separate channels, the reorganization layer rearranges the high-resolution feature maps from a lower layer. The performance of the layer of reorganization is fed to the layer of depth concatenation. This feed is bypassed and skipped many convolutional processes so that, as previously mentioned, the output is very fast. The concatenation layer of the depth concatenates the reorganized high-resolution features from a higher layer with the low-resolution features.

The purchased products are target objects and the system does not detect non-targets as we have previously done [17]. We believe that the system is robust enough to recognize targets. The selective detector strategy is the key to avoiding true-negatives (TN) and false-positives (FP) in the system. So that machine vision with precision to make decisions during manipulation and the time detection will be shortened. For instance, the detection results are provided to show bounding boxes with each confidence level on shelf environment. A detailed discussion of the shelf environment would be given in Section 4.

### 3.5. Localization for Recognized Products

**Figure 6:**
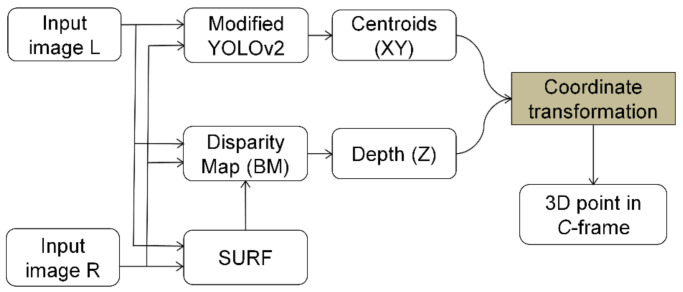
Flow diagram of the coordinate transformation.

Several segmented targets were generated for the purchased products, in which one segment depicted a detected target through deep learning with a modified selective YOLOv2 detector. The bounding boxes were calculated to get centroid points and depths of the consumer product frame *P* in the camera frame *C*. Our workflow in the estimation of depth is shown in Figure 6. The depths were extracted from the double check bounding box and the centroids were verified via SURF with a disparity map. Moreover, using the intrinsic camera parameters, the depths have been converted from the target *P*-frame to the *C*-Stereo camera-like.

**Figure 7:**
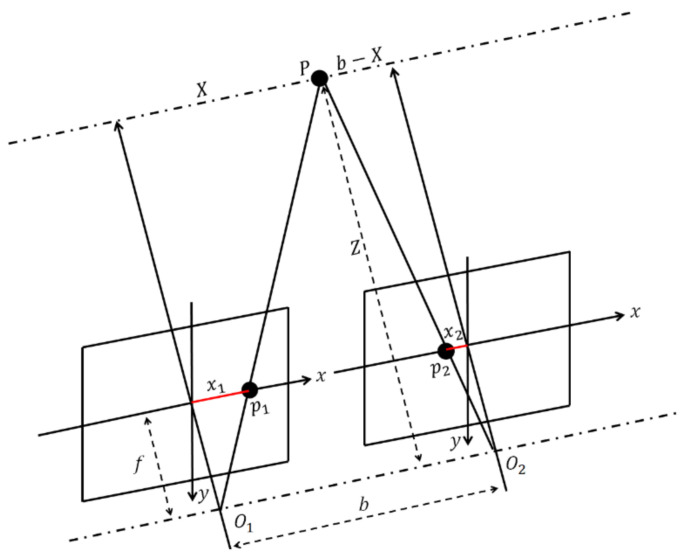
Triangulation scheme of stereo camera-like. The $O_{1}$ is camera plan 1 in the left side then shifting along baseline *b* and taken a picture in $O_{2}$ as camera frame of plan 2.

The configuration scheme of the stereo camera-like is based on stereo vision, shown in Figure 7. Mono camera set in parallel line *x* is fixed in the workspace and *O* as an optical center. The baseline b is constructed by shifting this camera with the focal length *f* along *x* line. The projections of a given reference point $P(X_{p},Y_{p},Z_{p})$ are $p_{1}(x_{1},y_{1})$ and $p_{2}(x_{2},y_{2})$ in the image plan 1 and image plan 2, respectively. In perspective projection, the image coordinate of *P* in two image planes is shown in Figure 7 to simplify the calculation [39].

In Figure 7, we assume just one camera only for defining the object. After camera shift along *b* line, both images are parallax on the shifted cameras, and the *Y* axis is perpendicular to the page. According to the theory of identical triangles, the Equations (1) and (2) are obtained, and Equation (3) shows the depth of *P* point, *Z*.

(1) $$\frac{X}{x_{1}}=\frac{Z}{f};\;\frac{X}{y_{1}}=\frac{Z}{f};\;\frac{b-X}{x_{2}}=\frac{Z}{f}$$

(2) $$b=\frac{Z}{f}x_{1}+\frac{Z}{f}x_{2}$$

(3) $$Z=\frac{b\times f}{x_{1}+x_{2}}$$

where *x*_1_ and *x*_2_ are the pixel locations on the 2D image. The disparity *d* is the difference of *x* coordinates in image 1 and image 2,

(4) $$d=x_{1}-x_{2}$$

After Z has been obtained, we can respectively obtain the *X* and *Y* coordinates of *P* point using equations in (5),

(5) $$X=\frac{Z\times x_{1}}{f};Y=\frac{Z\times y_{1}}{f};Z=\frac{b\times f}{d}$$

where *X*, *Y* and *Z* are the actual positions on the 3D image.

It is assumed that the products sold in offline shop are within the gripper’s reach. For this reason, the centroid position of each detection result needs to be searched. The easiest technique is to calculate the centroids from the bounding box expressed in (6) below:

(6) $$B_{box}=\left[\begin{array}{cccc} a_{11} & a_{12} & a_{13} & a_{14}\\ a_{21} & a_{22} & a_{23} & a_{24}\\ a_{31} & a_{32} & a_{33} & a_{34}\\ \vdots & \vdots & \vdots & \vdots \\ a_{n1} & a_{n2} & a_{n3} & a_{n4} \end{array}\right]$$

The bounding box matrix *B_box_* has four columns $a_{\left[1,\ldots ,4\right]}$ and the number of rows depends on the number of detected products $a_{\left[n,4\right]}$ on each shelf. So, we could find the centroid ($X_{\mathrm{cen}}Y_{\mathrm{cen}}$) from Equations (7) and (8).

(7) $$\left\{ \begin{array}{l} X_{c}=B_{box}(:,a_{11});Y_{c}=B_{box}(:,a_{12})\\ a=B_{box}(:,a_{13});b=B_{box}(:,a_{14}) \end{array}\right.$$

(8) $$\left\{ \begin{array}{l} X_{cen}=X_{c}+\frac{a}{2}\\ Y_{cen}=Y_{c}+\frac{b}{2} \end{array}\right.$$

From Equation (8), the centroid can be calculated and becomes the reference point for a gripper to pick the target. The centroid point in this condition is still in 2D image, so it is necessary to add *Z* value. The *Z* value is obtained from Equation (5) and verified by Equations (7)–(10).

#### 3.5.1. SURF with Disparity Map

**Figure 8:**
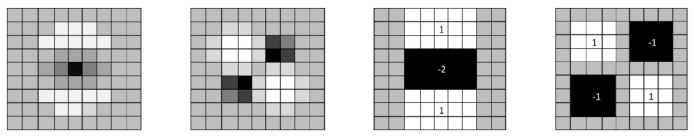
Left to right: The 9 × 9 box (discretized and cropped) Gaussian second order partial derivatives in *y*-direction. The grey regions are equal to zero.

In the traditional Speeded Up Robust Features (SURF) algorithm, the box filter is used to approximate the convolution of the 2D images [40,41]. The Gaussian second- order derivative can simplify the calculation and improve the efficiency [23]. The SURF detector uses Hessian matrix because of its good performance in computation time and accuracy. Given a point *X* = (*x*, *y*) in an image *I*, the Hessian matrix H(x,σ) in *x* at scale σ is defined as follows,

(9) $$H(x,\sigma )=\left[\begin{array}{cc} L_{xx}(x,\sigma ) & L_{xy}(x,\sigma )\\ L_{yx}(x,\sigma ) & L_{yy}(x,\sigma ) \end{array}\right]$$

where $L_{xx}(x,\sigma )$ is the convolution of the Gaussian second-order derivative $\frac{\partial^{2}}{\partial x^{2}}g(\sigma )$ with the image *I* at point *x*, and $L_{xy}(x,\sigma )$ and $L_{yy}(x,\sigma )$ have similar definitions. The 9 × 9 box filters in Figure 8 are approximations of a Gaussian with scale σ = 1.2 and represent the lowest scale for computing the blob response maps while denoted by ${\;D}_{xx},{\;D}_{yy},\;$and ${\;D}_{xy}.$ For computational efficiency, the weights applied to rectangular regions are kept simple as Equation (10),

(10) $$\det(H_{approx})=D_{xx},D_{yy}-{\left(0.9D_{xy}\right)}^{2}$$

To match the expression for the Hessian’s determinant and number of *w* = 0.9, the relative weight *w* of the filter responses is used. With this detector shown in Figure 8, the image to be detected must be converted into a gray image first. The matching points were increased to 100 points to boost precision, in the expectation that the mean precision would increase with a low standard deviation. The SURF alone, however, was not accurate enough. So, with another technique, namely the disparity map, is needed to search matching points.

The disparity map *D(x,y)* represents the displacement of the corresponding pixels between the left and right images. However, locating corresponding pixels is difficult in reality. Some variables may cause problems in the non-occlusion pixels, such as non-textured-homogeneous, repeated-texture, and camera noise [42,43,44]. The calculation of the disparity is done by block matching (BM) for all pixels and the validity of the disparity significance is defined as follows.

(11) $$D_{L\to R}(x,y)=\overset{\arg\min}{d\in [0,D_{\max}]\varepsilon_{L\to R}^{d}(x,y)}$$

(12) $$\varepsilon_{R\to L}^{d}(x,y)=\frac{\sum (u,v)\sum \in W|f_{r}(x-u,y-v)-f_{l}(x-u+d,y-v)|}{\sum (u,v)\sum \in W|f_{r}(x-u,y-v)+f_{l}(x-u+d,y-v)|}$$

**Figure 9:**
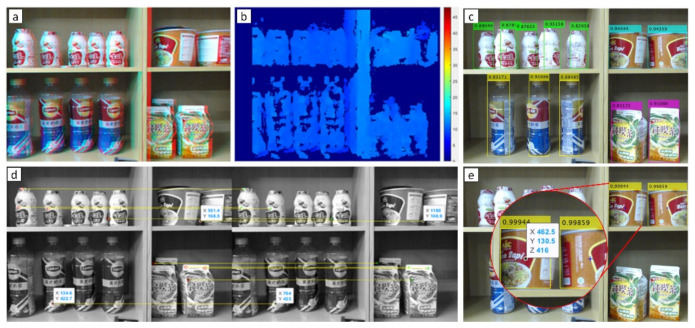
(**a**) Red-cyan composite view of the rectified stereo pair image, (**b**) block matching disparity map from (**a**), (**c**) confidence levels of original YOLOv2, (**d**) merger of left and right image with n = 10 matching points, and (**e**) output selected detector of modified YOLOv2 with confidence level with XYZ coordinates.

In Figure 9b, block matching is applied to the disparity map. The disparities between the left image and the right image are derived from (11) and (12), where $\varepsilon_{R\to L}^{d}(x,y)$ is the normalized block matching error with the horizontal disparity d, W is window of the block matching, and $D_{max}$ is the maximum value disparity within the permissible limit. The following will obtain the disparity from the right image $f_{r}$ to the left image frame $f_{l}$, to check the observed disparity, while *u* and *v* are the number of pixels in *xy*-camera image plane, respectively.

(13) $$D_{R\to L}\left(x,y\right)=\begin{array}{c} \arg\min\\ d\epsilon \left[-D_{max,}0\right] \end{array}\;\varepsilon_{R\to L}^{d}\left(x,y\right)$$

The minimum matching error (MME) then calculates how close the pair image values in the left image (*x,y*) are to and in the right image with disparity (*x* + *d,y*) to the identical points. The MME is known to be (14).

(14) $$MME\left(x,y\right)=\varepsilon_{L\to R}^{d}\left(x,y\right){|}_{d=D_{R\to L}\left(x,y\right)}$$

Figure 9 manifests the sequence of detection and localization process. In Figure 9a, the image looks shaded because it is a composite view between the left and right images with a baseline of 50 mm. Meanwhile, Figure 9b showed the result from the disparity map in Figure 9a by applying block matching Equations (11)–(14). When capturing pairs of objects, the image is separately used as YOLOv2 input for detection purposes, and the detection results are shown in Figure 9c. Before finally knowing the type of product and position in Figure 9e,d acts as a triangulator between stereo camera-like in Equation (5).

#### 3.5.2. A Half Shelf System

Due to the limited reach of the robotic manipulator, the store shelves need to be visually shortened in half; left and right. In Figure 2, the robotic manipulator will look for the target on the left side first; if it does not find the purchased product, it changes to the right side. The dimensions of the half shelf are 622 mm × 785 mm × 200 mm so that those of the whole shelve are 1244 mm × 785 mm × 200 mm.

Scanning the purchased products on the left and right depends on the data on purchase. The REST API reply becomes a trigger for this modified YOLOv2 architecture. If the purchased product has been found by modified YOLOv2 on the left shelf, the gripper immediately moves to hold it. On the other hand, if not found on the left shelf, the gripper shifts to the right shelf to detect until the gripping process occurs. Algorithm 1 illustrates this; if the purchased product *Pr* is in the first half of the shelf and the depth of camera world $Z_{C}$ is known, a matching process is carried out so that the depth of the world $Z_{R}$ robot will be determined.
**Algorithm 1** Search Purchased Product in A Half Shelf **predefined**: a product in a half shelf system frame; **input**: the REST API data-driven.
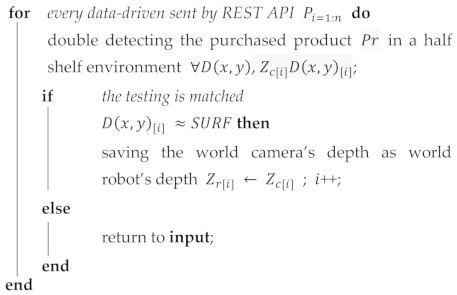


The shelf has three columns and three rows with a depth of 200 mm. Meanwhile, the position of sold products varies, generally in the range of 5–150 mm. This position is our challenge in conducting further verification of depth. The products displayed on the shelf have variations, as previously mentioned in Table 1. The largest volume is 230 mm × 120 mm × 62 mm with a maximum weight of 532 g; while the types of packaging include bottles, pouches, cups, cans, and carton boxes. We do not consider the target orientation because the position of *XY*’s target is always upright to the end-effector in this study.

#### 3.5.3. Shelf to World Transformation

**Figure 10:**
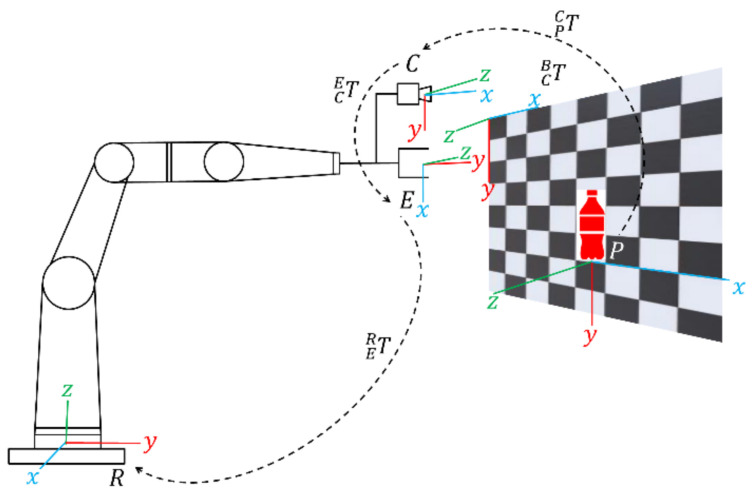
Eye-in-hand robot manipulator frame in an offline shop. The transformation begins from target in shelf coordinate frame *P* to camera coordinate frame *C*, then transforms from *C* to coordinate frame *E*, and continues to transform frame *R* as robot base.

The mono camera mounted to the end-effector perceives the product on the shop shelves in 2D coordinates. The coordinates of the purchased product in camera frame *C* need to be converted into those in the end-effector frame *E*. Figure 10 shows relationship among each frame, where *P* is the purchased products frame and *R* is the robotic base frame. Because a half shelf system is adopted, two transformations are required for each left and right shelf.

The transformations between frames perception downsizing into links of the MELFA RV-3SD robotic manipulator have been included in the robot controller. Let PR be the location of the purchased product *Pr* with respect to the *R*-frame and PC be the location of purchased product *Pr* in the *C*-frame. The transformation of the target coordinate from *P*-frame to *R*-frame could be expressed in (15).

(15) TRP=TCPTECTRE

where TRP is transformation from *P* frame to *R* frame, the target frame and the robotic base frame, respectively. The TCB depicted in Figure 10 can be obtained from camera calibration while TBR is known. Based on parameters of TCB and TBR, TCR could be obtained.

An offset of 280 mm is added to calculate the initial pose in the gripper frame based on the Robotiq three fingers offset configurations, to ensure that the camera aims the center of products at the initial pose. Robotic manipulator is designed to move from $O_{1}$ to $O_{2}$ baseline as initial position, picking process, and placing to home position *movh*←*gpc* until release the gripped product into a box.

## 4. Picking Algorithm for Offline Shop

The offline shop with a robotic manipulator needs to be aware of its shelf environment to avoid manipulator and gripper from knocking products and shelves. The robotic system also must verify the manipulation plan and accurately grasp the purchased products so that possible damage can be suppressed [45].

### 4.1. Purchased Products in Shelf

After the selector receives the driven data, it will immediately request the suitably selected YOLOv2 detector. The position of the product on the shelf is always upright, except for instant noodles and vermicelli. Therefore, the target orientation is not considered in this study. Besides, we do not take the various forms of product packaging into account because the gripper is adapted to the target.

Products in the shelf are not affected by the arrangement and combination of products in the same shelf. For example, a shelf containing two bottles of British Milk Tea, one yogurt, and one ABC Soy Sweet Ketchup, is acceptable to the system while there is a tolerance for grippers. The condition of the uniform products on the shelf within one camera frame allows multi-detection and overlapping to occur. Although the random combination causes multi-detection occurring, it is possible to suppress overlapping.

We deliberately bring up all detection results even though it can be selected according to the number of products purchased by consumers directly. For instance, a consumer bought two ABC Soy Sweet Ketchups, and then the detection results would appear only the first and second highest confidence values. The option brings up all detection results so that the robot can keep a safe position to grasp, and the safe position tends to avoid the edge of the shelf to prevent collisions.

**Figure 11:**
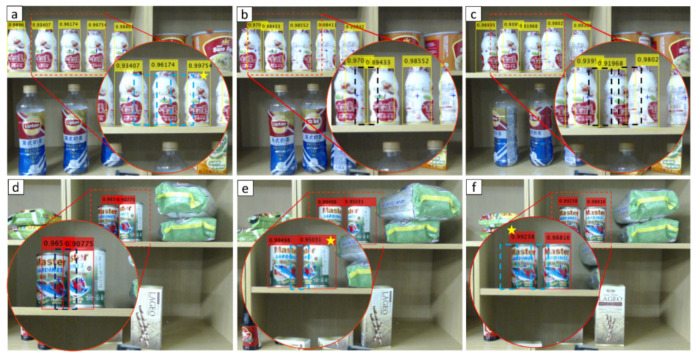
The possibility of detection on purchased products in one column of shelf; (**a**) multi-detected purchased products on inline layout and dashed-green line shown the safe narrow space for a gripper, (**b**) the overlapped bounding boxes of purchased products on inline layout, (**c**) the overlapped bounding boxes form overlapped products, (**d**) the overlapped bounding box on mixed products, (**e**) the overlapped bounding box on mixed products with yellow-star as a chosen to pick, and (**f**) the mixed products on one shelf with safe edge collision. In real space, the dashed-black line indicates it cannot be reached by the gripper and (**a**–**c**) as a left, while (**d**–**f**) as a right half shelf.

Figure 11 depicts the possibility of detection on the purchased products. Figure 11a shows the clear and safe position on the inline products for picking by the gripper. Meanwhile, if the products are too tightly positioned or not inline, there will be a chance that the bounding box overlaps, as shown in Figure 11b. Overlapping occurs due to slightly inaccurate and precise detection, but at the accepted limit, >0.85. In addition, overlapping can occur naturally because the products are physically arranged back to back (not inline), as shown in Figure 11c. Observing Figure 11d, there has been an overlapping bounding box. Figure 11b–d occur because the products are close together and/or have differences in-depth and Equations (6)–(8) are used to find the centroid of the bounding box, which is ultimately used as a localization reference. In Figure 11e, where the two product targets are the same and in a safe position for pick and place but have different confidence scores, the one with higher value will be grasped first using Algorithms 1 and 2 (stated in Section 4.2.3).
**Algorithm 2** Ascertain Whether Purchased Product Is Within Multi-Detection, Overlapping, or Mixed Products**Predefined** **input**$:\;\mathrm{coordinates}\;\mathrm{of}\;\mathrm{purchased}\;\mathrm{products}\;P_{i}$; $\mathrm{the}\;\mathrm{depth}\;\mathrm{of}\;\mathrm{purchased}\;\mathrm{products}\;Z_{i}.$ : **Algorithm 1**.**for***each detected purchased product*$P_{i=1}$$\;\mathrm{to}\;P_{i=n}$**do**
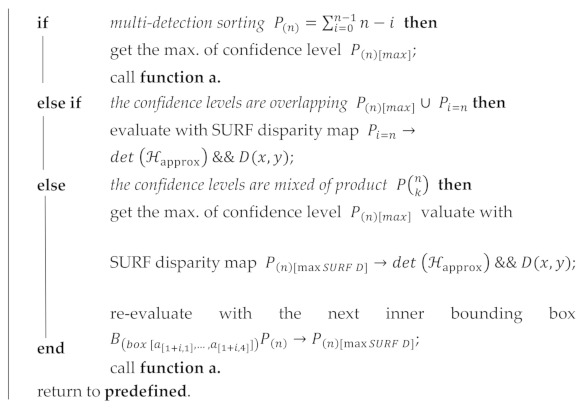
**end****function a**heading the gripper to the product $P_{\left(n\right)\left[max\right]}\leftarrow \exists \;p_{\left[x,y,z\right]}$; grasping the product $gpc\leftarrow P_{\left(n\right)\left[max\right]}$; placing to home position movh←gpc.

### 4.2. Grasping Purchased Product

#### 4.2.1. Multi-Detection

To identify purchased products in the safe shelf position shown in Figure 11, it is necessary to determine the distance between each product based on bounding boxes. Thereafter, it is important for the limited range of the gripper to 72 mm or 120 mm to make a decision. The distance between one product and another can be squashed so that the gripper fingers cannot enter to grasp. The worst risk is if the gap between the products is too narrow, the gripper fingers will hit the target or the other. If the gripper hits the target, the position of the purchased product at least will be shifted from its original position and the gripper potentially fails to grasp. As an illustration, there are five yogurts in a row detected as targets with each confidence value while the consumer bought one item only, then four yogurts must be eliminated. For the separation process to run well, the multi-detected results need to be sorted by $P_{\left(n\right)}=\overset{n-1}{\underset{i=0}{\sum }}\left(n-i\right)$ so that the maximum of confidence level $P_{\left(n)[\max\right]}$ is obtained.

However, as there were certain conditions in overlapped purchased products that caused the sorting production to surpass the safe gripper limit, we perceive that our approach was not always quite acceptable, as shown in Figure 11c. Empirically, the sorting method does not apply to overlapped purchased products. By applying this method might be rated as a non-optimal condition.

#### 4.2.2. Overlapping

Contrary to the first method, the purchased products that overlap by $P_{\left(n)[\max\right]}\cup P_{i=n}$ with the highest confidence level tend to be ambiguous make a grasping decision. For instance, suppose there are five yogurts on a shelf, where three are in the front line and the rest two in behind with the highest confidence level on one of two in behind. If the conditions are as above, the verification will be made using the SURF-Disparity Map and handled accurately.

To solve this overlapping problem, the centroid bounding box marked (+) is compared to the disparity map. However, the possibility of comparing two identical images has the potential to be missed, therefore SURF was applied and was able to identify overlapped purchased products.

#### 4.2.3. Mixed Products

In a mixed product by$\;P\left(\begin{array}{c} n\\ k \end{array}\right)$, one shelf can contain two or more types of products, as shown in Figure 11c. The position of Master Sardines next to British Milk Tea allows the bounding box to overlap with the same product types shown in Figure 11b. The advantage of using the selected YOLOv2 detector eliminates the possibility of true-negative results due to the error detection of other products that are not compatible with this detector.

It needs to pay attention to shelf space to solve the overlapping bounding box position in mixed products. One shelf cell covering an area of 414 mm × 261 mm × 200 mm contains two types of products, so it is only 207 mm for one product type. For this reason, it is impossible to rely on the highest confidence value as a benchmark to be picked by the gripper because the confidence value may occur in a product where is close to the shelf edge. The method is done concerning two centroids, and the position of the rightmost or leftmost centroid will be avoided by the system as written in Algorithm 2. In short, the system tends to choose the inner bounding box by $B_{\left(box[a_{[1+i,1],\ldots ,}a_{\left[1+i,4\right]}]\right)}P_{\left(n\right)}$ rather than simply the highest score.

Although the weakness of multi-detection is resolved in the overlapping matters, this method also has disadvantages because of collapsed products. A collapsed product can still be recognized by the system but cannot be grasped because the gripper finger has a limited range. Furthermore, the performance of two stratified methods is a double check by conducting rules. Not only do multi-detection and overlapping have potential problems, but mixed products also have the potential to overlapping problem that can be resolved by Algorithm 2. Finally, by double-checking, the system can solve localization problems through those methods.

## 5. Experimental Works

### 5.1. Experimental Settings

In this study, we carried out a scenario that the system was run for SME as much as possible, which the cost of the devices was reduced while still paying attention to system performance. We hope that this system could be adopted cheaply with the minimum specifications as shown in Table 2. The reach of the manipulator robot is limited to a radius of 642 mm and positioned fixedly on the table. It is necessary to arrange the shelf in both the number and the position as described in Section 3.5.2. This study is also proved with a video available in this link https://youtu.be/WD1mIL7o8X8 (accessed on 28 November 2020).

### 5.2. Evaluation of Detection Method

As defined in Equation (16), the detection results of confidence, accuracy, precision, recall, performance (F1), average precision (AP), average *µ*, standard deviation *σ* and time are evaluated. The numbers of true-positive (TP), false-positive (FP), true-negative (TN), and false-negative (FN) were included in roughly 500 recorded images in total to testify the entire detection evaluation process. To compute the precision, recall, F1 score, and AP, a confidence value of 0.85 was set [46]. Starting from observing, TP satisfies the detection with an IoU value > 0.5 and no double bounding boxes, and the opposite condition is called FP. While the true negative (TN) criterion is that there are no double bounding boxes and the IoU value is <0.5, the rest is FN if it is wrongly detected and the IoU value is <0.5 or even there is no bounding box.

(16) $$\begin{array}{l} accuracy=\frac{TP+TN}{TP+TN+FP+FN}\\ precision=\frac{TP}{TP+FP}\\ recall=\frac{TP}{TP+FN}\\ F_{1}=\frac{2\times precision\times recall}{precision+recall}\\ AP=\int_{0}^{1}p(r)dr \end{array}$$

The results could be seen in Table 3 with each product code A (Indomie Soto Instant Noodle), B (British Milk Tea), C (Golden Apple Yogurt), D (Tai Lemon Tea), E (ABC Soy Sweet Ketchup), F (Master Sardines), G (Lageo Wafers), H (ABC Cup Baso Noodle), and I (Bun Gao Vermicelli). The highest value for confidence is H, while accuracy, precision, recall, F1, and AP are in a product I. The best detection speed is for product of C with µ = 0.053 with σ = 0.001. The comparison of results by our proposed method with MobileNetv2 and ResNet18 are listed in Table 3, and the bolds are the highest values for each parameter.

### 5.3. Experiments of AIoT for grasping

In Table 4, we compare the performance between the YOLOv2 detector and our modified YOLOv2 detector with varying amounts of combination. We grasp purchased product $gpc\leftarrow P_{\left(n)[\max\right]}$ on the shelf models using 88 grasp attempts in our setting, to evaluate the effective performance of the method through the data of consumer purchase process. Table 4 shows the evaluation of the two methods, where each method is evaluated for two conditions so that there are a total of four combinations, 8–10 grasp attempts each based on online purchase. Each trial product position on the shelf is always changed randomly, including on the right or left shelves. Besides, the success rate in holding effort is substantially slightly increased, from 0.807 original YOLOv2 to 0.835 after we modified the YOLOv2 detector.

Moreover, these results could be informative to understand the performance of the modified selective YOLOv2 detector. The data-driven mode plays an important role in this technique. First, raw data from the REST API reply become the key to determine the selection process and then call the detector type after data parsing. Second, the relatively short response time of the IoT system (µ < 0.401 s) brings the opportunity for AI to double-check for the same frame so that localization can be improved. The grasp failure was due to the gripper that we could not control the finger opening, most of these grasp failures were the product width close to the gripper finger opening width. The gripper that we use is adaptive to shape but restricted to a couple of choices; straight open (72 mm) and wide-open (120 mm) types, as well as this limits the size of the product we could hold.

About 80% of systems are successful in grasping, even though the localization results are very accurate. After being analyzed, the narrow straight-open gripper causes the potential purchased product to be pushed-in when grasping a target so that the XYZ position changes. As an illustration, product of B (British Milk Tea) has a width of 64 mm while the straight-open gripper is only 72 mm, meaning that there is only a tolerance of 4 mm for the left and right sides as shown in Figure 9e. The best way is to use a vacuum gripper, but it has weaknesses in holding products with uneven surfaces, easily shifted positions, and plastic bags such as products: A (Indomie Soto Instant Noodle), C (Golden Apple Yogurt), E (ABC Soy Sweet Ketchup), H (ABC Cup Baso Noodle), and I (Bun Gao Vermicelli).

### 5.4. AIoT Shop in Society 5.0 Evaluation

AIoT Shop evaluation involves integrating an integrated manner of three parts of the evaluation: AI, IoT, and online shop. From each of these parts, several indicators are derived. The metrics evaluated in the AI segment include precision, accuracy, recall, and performance aspects. Besides, the IoT assessment is focused on Asghari et al. [47,48,49]. Indicators include security, response time, cost, and consumption of energy (CoE). The online shop with the app version and the website is evaluated online by the Website Grader Tool which includes four indicators: performance, search engine optimization (SEO), mobility, and security.

**Figure 12:**
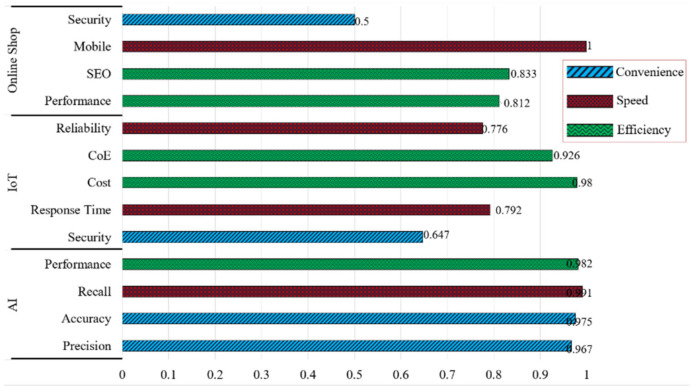
The clustered bar of AIoT shop evaluations. The range of each value locates between 0 and 1 from three aspects with 13 indicators of Society 5.0 context.

Broadly, this AIoT shop evaluation in Society 5.0 touches upon three terms: efficiency, speed, and convenience. For this reason, Figure 12 depicts the correlation of each aspect that has been evaluated in the context of Society 5.0. Figure 12 also shows that the AI system built with modified YOLOv2 can work very well with a score of >0.95, slightly moderate for IoT µ > 0.82, superior in terms of cost but quite risky in terms of security. Even with online shop among an absolute score it has a high score on the mobile side, while on the contrary, only 0.5 for the security indicator. Overall evaluation of the AIoT shop in the context of convenience, speed, and efficiency reaches a score of 0.860.

We took AI testing from the data in Table 2. While the online shop test was obtained from the Website Grader Tool with the specifications of the online shop page size was light 1.7 MB of 3 MB and with a page load speed, 4.2 s of 5.3 seconds’ maximum limit. From the security side, we have used HTTPS or SSL and the system suggests updating JavaScript libraries. Finally, the IoT evaluation is based on [47] at a low cost. In 2020, the cost of IoT devices is only USD 0.38 while this system does not require other sensors and just connects the 6-DOF robotic manipulator to internet.

## 6. Conclusions

This study intends to develop a picking algorithm using AIoT and implement the results of the algorithm at convenience shops to follow the trend of Society 5.0. The picking algorithm is based on YOLOv2 modified into a parallel detector through a data-driven mode. A robotic manipulator in an offline shop is connected to the internet for picking off the shelf. To improve localization accuracy, we use a stereo camera for every purchased product which is tested twice and verified using a SURF Disparity Map. The test results show that the picking algorithm with AIoT correctly takes the purchased products with a success rate of 0.835 in settings such as in a convenience shop. From the indicator of time per transaction, the service speed is at rate of 0.792.

The results of the developed picking algorithm are applied to a limited extent for rack-to-box picking. Our next study will involve an automated guided vehicle (AGV) for delivery from offline stores to consumers by adopting several localization strategies.

## Figures and Tables

**Table 1:** The characteristics of the products sold in our shop.

Products	Title 2		Title 3	
	Size (mm)	Package	Weight (gr)	Shape
ABC Soy Sweet Ketchup	64 × 42 × 42	bottle	140	S
British Milk Tea	222 × 67 × 67	bottle	532	S
Bun Gao Vermicelli	120 × 220 × 62	pouch	200	B
Cup Noodle ABC	92 × 107 × 84	cup	65	C
Golden Apple Yogurt	106 × 52 × 52	bottle	175	S
Indomie Soto Noodle	130 × 92 × 32	pouch	76	B
Lageo Wafers	150 × 71 × 34	box	47	B
Master Sardines	86 × 52 × 52	can	176	S
Tai Lemon Tea	144 × 70 × 71	box	255	B

**Table 2:** The minimum settings of our system.

Parameters	Specification
CPU	Intel Core i5 @3.0 GHz (6 CPUs)
Memory	RAM 16 GB
Op. System	Windows 10
GPU	Onboard Intel UHD Graphics 630
Camera	Logitech C920
Robot Arm	MELFA RV-3SD 6 DOF
Gripper	Robotiq 3 fingers
IDEs	MATLAB, PHP 7, Laravel
Database	MySQLi
Host	https://indoaltantis.com (1 GB)
Mobile Comp.	Android 6+, iOS
Browser Comp.	Chrome (recommended), Firefox, Opera, Microsoft Edge, and Safari

**Table 3:** Grasping evaluation of detection using a modified YOLOv2 and another two.

Class of Product	Method	Parameters						
		Confid.	Accur.	Precis.	Recall	F1	AP	Time (s)
A	modYOLOv2	**0.942**	**0.990**	0.990	**1**	0.995	**0.990**	**0.055**
	MobileNet2	0.905	0.963	0.963	**1**	0.981	0.941	0.157
	ResNet18	0.890	0.950	**1**	**1**	**1**	0.961	0.056
B	modYOLOv2	**0.938**	**0.972**	0.971	**1**	0.985	0.927	**0.054**
	MobileNet2	0.626	0.925	**1**	**1**	**1**	0.958	0.053
	ResNet18	0.707	0.750	0.944	**1**	0.971	**0.971**	0.056
C	modYOLOv2	**0.852**	**0.990**	**1**	**0.989**	**0.994**	**0.771**	**0.053**
	MobileNet2	0.768	0.740	**1**	0.958	0.978	0.827	0.087
	ResNet18	0.719	0.750	0.969	0.969	0.969	0.633	**0.053**
D	modYOLOv2	**0.925**	**0.990**	**0.990**	**1**	**0.995**	**0.990**	0.054
	MobileNet2	0.864	0.963	0.963	**1**	0.981	0.909	**0.053**
	ResNet18	0.862	0.925	0.974	**1**	0.987	0.923	**0.053**
E	modYOLOv2	**0.885**	0.854	0.854	**1**	0.921	0.754	0.053
	MobileNet2	0.818	0.777	0.777	**1**	0.875	0.650	0.054
	ResNet18	0.709	**0.875**	**0.875**	**1**	0.933	0.739	**0.052**
F	modYOLOv2	**0.922**	**0.981**	**0.981**	**1**	**0.990**	**0.979**	0.054
	MobileNet2	0.708	0.963	0.963	**1**	0.981	0.998	0.053
	ResNet18	0.690	0.875	0.875	**1**	0.933	0.739	**0.052**
G	modYOLOv2	**0.875**	0.945	0.990	0.954	0.971	0.867	**0.055**
	MobileNet2	0.641	0.851	**1**	0.851	0.920	0.776	0.058
	ResNet18	0.785	**0.975**	0.975	**1**	**0.987**	**0.957**	0.056
H	modYOLOv2	**0.952**	**0.981**	**1**	**0.981**	**0.990**	0.907	**0.056**
	MobileNet2	0.841	0.851	1	0.923	0.960	0.749	0.062
	ResNet18	0.952	0.925	0.974	1	0.987	**0.925**	0.058
I	modYOLOv2	**0.917**	**1**	**1**	**1**	**1**	**1**	0.056
	MobileNet2	0.868	0.925	1	1	1	0.9616	**0.053**
	ResNet18	0.742	0.950	0.974	0.974	0.974	1	0.055
µ modYOLOv2		**0.912**	**0.967**	**0.975**	**0.992**	**0.982**	**0.909**	**0.054**
µ MobileNet2		0.782	0.884	0.963	0.970	0.964	0.863	0.070
µ ResNet18		0.784	0.886	0.951	0.994	0.971	0.872	0.055
σ modYOLOv2		**0.032**	**0.043**	0.044	0.015	**0.015**	**0.089**	**0.001**
σ MobileNet2		0.097	0.079	0.068	0.049	0.039	0.112	0.032
σ ResNet18		0.089	0.079	**0.043**	**0.012**	0.022	0.124	0.002

**Table 4:** The modified YOLOv2 test evaluation.

Methods	Conditions	Num. of Successes	Num. of Failures	Success Rate	Ave. Rate
YOLOv2	Single product	74	14	0.841	0.807
	Mixed product	69	19	0.773	
Modified YOLOv2	Single product	77	11	0.875	0.835
	Mixed product	70	18	0.795	

## Data Availability

Not applicable.

## Supplementary Materials

The following are available online at https://youtu.be/WD1mIL7o8X8 (accessed on 28 November 2020). Video tittle: An IoT-Online Shop Collaborating with Robot Manipulator (MELFA RV-3SD).
